# Microglial M2 Polarization Mediated the Neuroprotective Effect of Morroniside in Transient MCAO-Induced Mice

**DOI:** 10.3389/fphar.2021.784329

**Published:** 2021-11-19

**Authors:** Hao Liu, Mei-Xian Ou, Qiao-Qiao Han

**Affiliations:** ^1^ Translational Medicine Center of Pain, Emotion and Cognition, Ningbo Key Laboratory of Behavioral Neuroscience, Zhejiang Provincial Key Laboratory of Pathophysiology, Ningbo University School of Medicine, Ningbo, China; ^2^ Shanghai Engineering Research Center of Phase I Clinical Research & Quality Consistency Evaluation for Drugs & Central Laboratory, Shanghai Xuhui Central Hospital, Shanghai, China; ^3^ Department of Immunology and Microbiology, State Key Laboratory of Oncogenes and Related Genes, Shanghai Institute of Immunology, Shanghai Jiao Tong University School of Medicine, Shanghai, China

**Keywords:** morroniside, interleukin-10, M2 polarization, middle cerebral artery occlusion, ischemic stroke

## Abstract

Morroniside, a secoiridoid glycoside from *Cornus officinalis*, is a class of small molecule non-peptide glucagon-like peptide-1 receptor (GLP-1R) agonists and possess many important biomedical functions. Our previous studies reported that GLP-1R agonist exenatide promoted M2 polarization and the expression of cell-specific anti-inflammatory factor interleukin-10 in neuropathological pain model. In this study, we proved that morroniside not only induced M2 polarization and stimulated interleukin-10 expression specifically in cortical primary microglia by p38β mitogen-activated protein kinases pathway but also protected nerve cells against H_2_O_2_-induced cell oxidative damage and prohibited ischemic injury by reducing infarct size, which is at least in part mediated by enhanced expression of microglial interleukin-10. In the cortical penumbra area in middle cerebral artery occlusion (MCAO) mice. In general, our results indicated that GLP-1R agonist morroniside might play a neuroprotective effect by inducing M2 polarization, and cyclic-AMP/protein kinase A/p38β pathway might mediate morroniside-induced expression of interleukin-10 protein in M2 microglia.

## Introduction

Transcriptomic analysis revealed that 75% of differentially expressed genes in ischemic brain tissues were derived from microglia, mainly related to inflammatory factors, cell activity, differentiation and metastasis, and so on (Khan et al., 2017). Thus regulation of microglia is an important direction for the development of targeted therapeutic drugs. Microglial activation is closely related to secondary brain damage induced by ischemic stroke, and its activation can be roughly divided into pro-inflammatory M1 type and anti-inflammatory M2 type, which can be induced to M2a type by interlukin-4 and interlukin-13, or to M2c type by interlukin-10 (IL-10) and glucocorticoids. The latter M2c type is importantly related to neuroprotection and tissue remodeling (Qin et al., 2019). The main treatment strategies for microglial activation in ischemic stroke included 1) directly inhibiting microglial activation (however, the drugs and related mechanisms of this strategy were diverse, with huge differences in efficacy) and 2) inducing M2 polarization classification, contributing to the repair of damaged tissues and the resistance of neuronal apoptosis (Kronenberg et al., 2018; Han et al., 2020).

Our previous studies demonstrated that a glucagon-like peptide-1 receptor (GLP-1R) agonist exenatide promoted M2 polarization and the expression of cell-specific anti-inflammatory factor IL-10 through cyclic-AMP (cAMP)/protein kinase A (PKA)/p38β pathway in neuropathological pain model (Wu et al., 2017a; Wu et al., 2018a; Wu et al., 2018b). In addition, we also found that morroniside, a secoiridoid glycoside from *Cornus officinalis*, was a class of small molecule non-peptide GLP-1R agonists and attenuated mechanical allodynia and thermal hyperalgesia by both systemic and intrathecal administration, which were completely blocked by pretreatment with intrathecal exendin (9-39), a classic GLP-1 receptor antagonist (Xu et al., 2017). Its analgesia in spinal nerve ligation–induced neuropathic pain was further proved to be related to the inducible expression of IL-10 and beta-endorphin, which could be reversed by primary antibodies of IL-10 and beta-endorphin in primary cultured microglia (Tang et al., 2020). In current studies, the mechanism of morroniside against ischemic stroke was also elucidated as prohibiting neural apoptosis and MMP2/9 expression, enhancing angiogenesis and improving microvascular functional integrity of the neurovascular unit after cerebral ischemia (Sun et al., 2014; Liu et al., 2016; Zeng et al., 2018).

This study intended to systematically reveal the promotion of M2 polarization and the enhancement of IL-10 expression by morroniside, and evaluate the pharmacodynamic effect of morroniside in a cell model induced by oxidative damage and a mouse model induced by ischemia/reperfusion.

## Methods and Materials

### Chemicals

Morroniside was commercially purchased from Chengdu Push BioTechnology Co. (Chengdu, China). Exendin (9-39) was bought from Shanghai TASH Biotechnology Co. (Shanghai, China). SB203580 and U0126 were obtained from Selleck Chemicals (Houston, TX, United States), while SP600125 was from Sigma Aldrich (St. Louis, MO, United States). Morroniside was diluted in phosphate buffered saline (PBS buffer, pH 7.5) or normal saline in cellular test and animal study, respectively. SB203580, U0126, and SP600125 were dissolved in DMSO/PBS (v/v, 1/4).

### Animals

Specific pathogen-free Swiss mice (male, 8–9 weeks) were ordered from the Ningbo Experimental Animal Institute in Ningbo University (Ningbo, China), in conformity with the animal care guidelines of NIH (Bethesda, MD, United States). The mice were reared in cages with free access to food and water following with the animal care guidelines of NIH (Bethesda, MD, United States). The animal protocols were approved by the Animal Care and Welfare Committee of Ningbo University (Ningbo, China) and conducted in compliance with the Guide for Care and Use of Laboratory Animals, and the Animals in Research: Reporting *In Vivo* Experiments (ARRIVE) guidelines (Kilkenny et al., 2011; Zhang et al., 2019).

### Isolation of Primary Cells

Cerebral cortex was removed from postnatal pups within 24 h, digested and dissociated in 0.05% trypsin for 30 min. The dispersive cells were neutralized with 5 ml complete Dulbecco’s Modified Eagle’s medium (DMEM) and centrifuged (500–600×*g*) for 8–10 min before resuspension with 1 ml manual pipette. For the microglial culture, cell suspensions were placed in a small cell culture flask (1 × 10^7^ cells/flask) and maintained in a 5% CO_2_ incubator at 37°C. Eight days later, microglial cells were prepared as floating cells by shaking the flask at 260 rpm for 2 h at 37°C. The harvested microglial cells were determined as Iba-1-positive cells (purity >95%) by immunostaining technology.

### The Ischemic Mouse Model Induced by Middle Cerebral Artery Occlusion

Transient MCAO model was executed for 90 min along with subsequent reperfusion for 24 h according to our former description (Liu et al., 2019). The mouse was anesthetized intraperitoneally with 1.5% pentobarbital sodium before surgery. The common carotid artery and the upper bifurcation site with internal carotid artery and external carotid artery were exposed after blunt separation. A commercial filament coated with a silicone tip was inserted into the common carotid artery for about 0.9 cm starting from the bifurcation site. The middle cerebral artery was blocked for 90 min at 33°C and withdrawn for subsequent reperfusion for 24 h. The successful rate of MCAO model was about 60%. All ischemic mice were randomly divided into each group (*n* = 4–6). The mice in the sham group were subjected to a similar surgery without inserting the filament into the common carotid artery.

The average infarct volume of cerebral slices (2 mm thick) was assessed by 2,3,5-triphenyltetrazolium chloride (TTC) staining. The fresh slices were incubated in PBS (pH 7.4)-buffered 1% TTC at 37°C for 15 min. The infarcted area was white while the uninfarcted part was rose colored. The average infarct volume of all four consecutive sections in two sides was calculated by the formula as follows: percentage (%) of hemispheric infarct = (contralateral hemispheric area − uninfarcted area of ipsilateral hemisphere)/whole spherical area × 100.

### Intracerebroventricular/Intravenous Injection in Mice

After MCAO surgery, the mouse was fixed in a stereotaxic instrument (RWD Life Science, Shenzhen, Guangdong, China). A microinjection syringe (10 μl; Shanghai Gaoge, Shanghai, China) was inserted into the hole drilled on the exposed skull above the right lateral ventricle (1.0 mm anteroposterior from bregma; 0.8 mm lateral; 2.5 mm in depth). Then the intracerebroventricular administration of 5 μl morroniside in different doses (300, 500, and 1,000 μg) or vehicle (saline) was injected slowly for 5 min, and the microinjection syringe was moved away after sustaining the needle tip in the hole for an additional 5 min to avoid backflow. The burr hole was sealed with a short injector tip and fixed with dental cement for multiple treatments. To determine the mRNA level of IL-10 on day 3 in MCAO mice, morroniside was administrated multiple times by intracerebroventricular injection twice a day, and the last treatment was 1 h before sacrifice.

To evaluate the pharmacodynamic effect of morroniside by peripheral administration, 40 mg/kg morroniside was administered at 0 h after surgery by single intravenous injection. Subsequently, the saline or GLP-1R antagonist exendin (9-39) (2 μg) was intracerebroventricularly administered in the same way as mentioned previously in the side of ipsilateral hemisphere. The effective evaluation was performed at 24 h after ischemic onset by TTC staining.

### Real-Time Quantitative Polymerase Chain Reaction

Total mRNA in primary microglial cells or cerebral cortex of the mouse subjected to transient MCAO was extracted and reversely transcribed by using a ReverTraAce qPCR RT-kit (Toyobo, Osaka, Japan) (Ouyang et al., 2014). Real-time quantitative polymerase chain reaction (RT-qPCR) amplification was operated in a Mastercycler ep realplex (Eppendorf, Hamburg, Germany) and Realmaster Mix (SYBR Green I) (Toyobo, Osaka, Japan) was used as fluorescent marker to quantify cDNA. The real-time PCR primers are listed in Table 1. The melting curves were checked for the specificity of qPCR amplification. The quantitative analysis was calculated by using the 2^−ΔΔCt^ method after normalizing to mRNA level of GAPDH.

**TABLE 1:** The sequence list of mRNA primers for real-time PCR

CD68	Forward: 5′-CTC​TCT​AAG​GCT​ACA​GGC​TGC​T-3′
	Reverse: 5′-TCA​CGG​TTG​CAA​GAG​AAA​CA-3′
iNOS	Forward: 5′-CTT​TGC​CAC​GGA​CGA​GAC-3′
	Reverse: 5′-TCA​TTG​TAC​TCT​GAG​GGC​TGA-3′
Arg-1	Forward: 5′-CGC​CTT​TCT​CAA​AAG​GAC​AG-3′
	Reverse: 5′-CCA​GCT​CTT​CAT​TGG​CTT​TC-3′
CD206	Forward: 5′-CCT​TAC​TGG​GCA​ATG​CAA​AT-3′
	Reverse: 5′-TGC​AAT​GGA​CAA​AAT​CCA​AA-3′
IL-4	Forward: 5′-ACA​GGA​GAA​GGG​ACG​CCA​T-3′
	Reverse: 5′-GAA​GCC​CTA​CAG​ACG​AGC​TCA-3′
IL-10	Forward: 5′-CTA​ACG​GAA​ACA​ACT​CCT​TG-3′
	Reverse: 5′-GAA​AGG​ACA​CCA​TAG​CAA​AG-3′
GAPDH	Forward: 5′-CCA​AGG​TCA​TCC​ATG​ACG​AC-3′
	Reverse: 5′-TCC​ACA​GTC​TTC​TGA​GTG​GC-3′

### Immunofluorescence Staining in Cerebral Sections

Cell-specific expression of GLP-1R in cerebral cortex: The cerebral sections (30 μm thickness) were prepared by frozen section technology. GLP-1R was double immunolabeled with microglia, astrocytes, or neurons (anti-GLP-1R, anti-glial fibrillary acidic protein (GFAP), anti-ionized calcium binding adaptor molecule 1 (Iba-1), and anti-neuronal nuclei (NeuN)) in the sections of cerebral cortex, which were visualized under a TCS SP8 confocal microscope (Leica Microsystems, Wetzlar, Germany) as described previously (Liu et al., 2019). The cerebral sections were baked in an air incubator at 60°C for 30 min to repair the tissue antigens, then washed with 0.05 M PBS (3 × 8 min) and incubated in sealing fluid (10% goat serum (v/v) containing 0.5% Triton X-100 (v/v)) for 1 h and subsequently in the anti-GLP-1R marker (1:100, Ab119287; Abcam, Cambridge, United Kingdom) and other primary antibodies (Iba-1 for microglia: 1:100, mouse monoclonal, Millipore Cat# MABN92; GFAP for astrocytes: 1:200, mouse monoclonal, Millipore Cat# IF03L-100UG; NeuN for neurons: 1:100, mouse monoclonal, Millipore Cat# MAB377) for additional 18 h at 4°C. After 0.05 M PBS washing (4 × 8 min), the double immunostaining of GLP-1R and each cytological marker was labeled with the Alexa-555-conjugated secondary antibody (1:200, goat anti-rabbit, Invitrogen Cat# Z25305) and Alexa-488-conjugated secondary antibody (1:200, goat anti-mouse, Invitrogen Cat# R37120) for 1–1.5 h at room temperature, respectively.

The fields localized within the cortical penumbra area were selected to quantify the immunofluorescent intensity of Iba-1/GLP-1R, GFAP/GLP-1R, and NeuN/GLP-1R colocalization, under a confocal microscope with ×40 magnification. The immunofluorescent intensity of positive staining area was included randomly, and the colocalized pixels in the merged images were measured following the solid conditional configuration. The image analysis (ImageJ Software, Wayne Rasband, NIH, United States) was conducted blindly by an investigator. The ratio of colocalized immunostaining area to total area was averaged from four sections of each sample (Wu et al., 2017a; Wu et al., 2018a; Liu et al., 2019).

### Western Blot

The proteins of cellular sample in this test were extracted and analyzed by Western blot technology (Liu et al., 2019). Primary antibody anti-phospho-p38 (1:1,000, Novus Biologicals Cat# NBP1-84305) was used for target protein with β-actin antibody (1:5,000, Santa Cruz Biotechnology Cat# sc-47778) as reference protein. After incubated with second antibodies, the protein bands were scanned by the Odyssey Infrared Imaging system (Li-Cor Biosciences, United States), followed with quantification of the gray intensity by ImageJ software (ImageJ Software, Wayne Rasband, NIH, United States).

### Cell Viability

A CCK-8 assay kit (Beyotime Institute of Biotechnology, Jiangsu, China) was applied to detect cell viability in N9 microglial cells. A portion of cells (5 × 10^3^ cells/ml) was incubated with complete DMEM in 96-well plates and cultured for 2 days. Hydrogen peroxide was mixed with DMEM to a final concentration of 600 μM for 15-min incubation. Subsequently, cells were washed with PBS and treated with 1 mM morroniside in the presence or absence of 10 nM exendin (9-39) for 12 h at 37°C. Finally, CCK-8 reagent was added into each well and incubated with N9 microglial cells at 37°C for 2–3 h accordingly. OD_450nm_ was measured by an EnSpire 2300 Multimode Plate Reader (Perkinelmer Co., United States).

### Statistical Analysis

Shapiro–Wilk normality test was performed to assess data distribution. Data with normal distribution were expressed in the form of means ± SEM, and statistically analyzed by using unpaired and two-tailed Student *t*-test or one-way ANOVA followed by *post hoc* Student–Newman–Keuls test. The statistical significance was defined as *p* <0.05. All statistical analyses were applied by using GraphPad Prism 7 (GraphPad Software, Inc., United States).

## Results

### GLP-1R Was Highly Expressed in Microglial Cells

**FIGURE 1:**
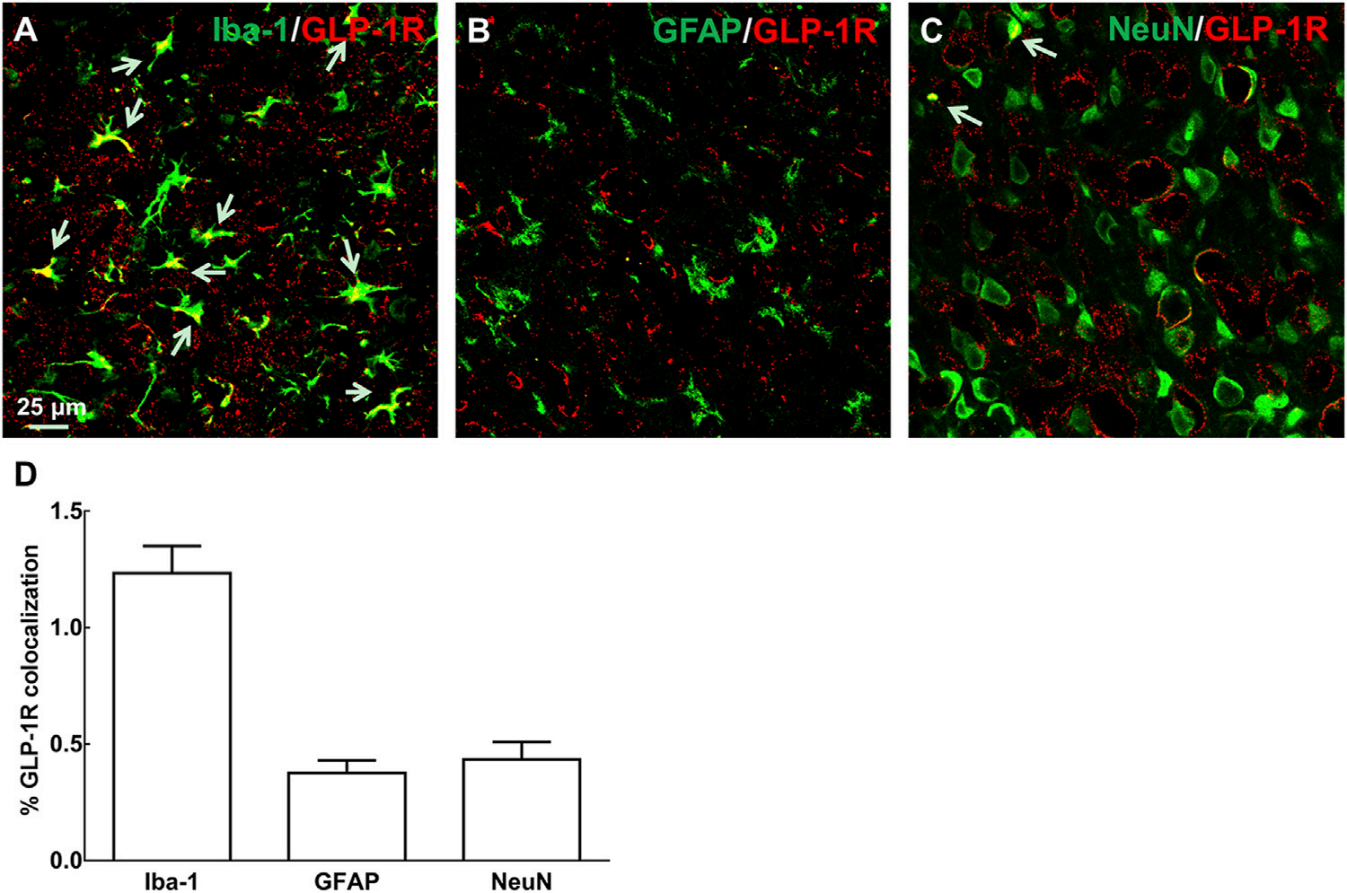
Immunological co-localization of GLP-1R with each cytological marker [**(A–C)**: anti-GFAP for astrocytes, anti-Iba-1 for microglia, and anti-NeuN for neurons, *n* = 4] on day 3 after ischemic onset in MCAO-induced mouse. It indicated that GLP-1R was mainly expressed in microglial cells with a small amount of fluorescent signal in neurons. **(D)** Fluorescent co-localization of GLP-1R with each cytological marker was quantitatively analyzed in the representative photomicrographs of cortical peri-infarct area in ischemia/reperfusion-induced mice at ×40 magnification (scale bar: 25 μm).

Immunological co-localization of GLP-1R with each cytological marker (anti-GFAP for astrocytes, anti-Iba-1 for microglia, and anti-NeuN for neurons) was performed on day 3 after ischemic onset in MCAO-induced mice (Figures 1A–C). The quantitative analysis of fluorescent immunostaining indicated that GLP-1R was mainly expressed in microglial cells with a small amount of fluorescent signal in neurons (Figure 1D). This result was in line with our former studies based on N9 microglial cells or neuropathic rodent model (Gong et al., 2014a; Wu et al., 2017b; Xu et al., 2017).

### Morroniside Induced M2 Polarization and Stimulated IL-10 Expression Specifically in Cortical Primary Microglia

**FIGURE 2:**
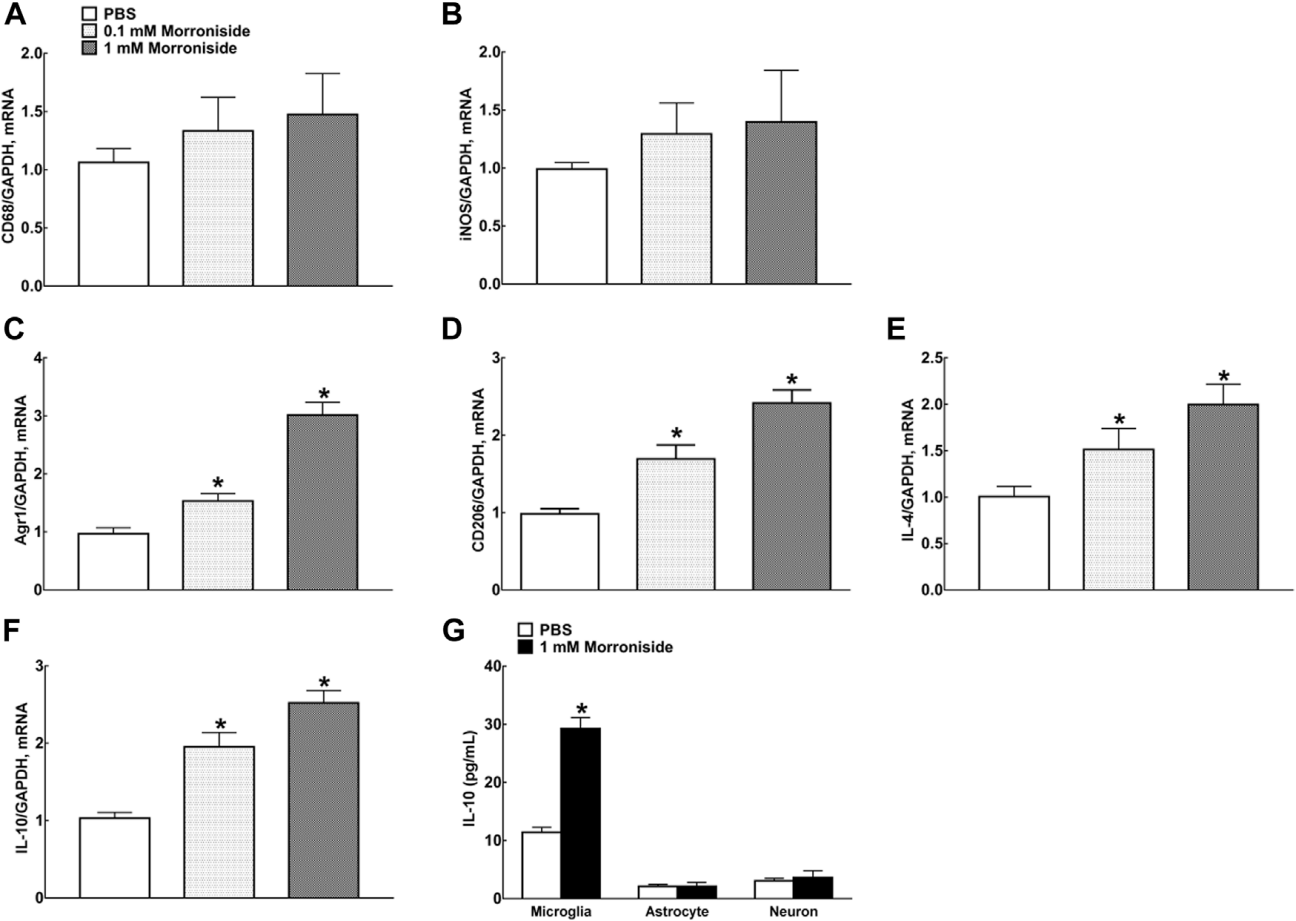
Morroniside significantly promoted the expression of M2 polarization–related proteins in primary microglia. **(A, B)** Morroniside did not significantly change mRNA levels of marker proteins CD68 and iNOS for M1-type microglia. **(C–F)** Morroniside significantly facilitated mRNA levels of marker proteins Agr1, C206, IL-4, and IL-10 for M2-type microglia. **(G)** ELISA was executed to detect the effect of morroniside on the expression of IL-10 protein in different types of primary cells (*n* = 3). The cellular experiments were repeated triply and all data were shown as mean ± SEM. *Denoted statistical significance compared with control group (*p < 0.05 by using one-way ANOVA and *post hoc* Student–Newman–Keuls tests).

Our previous study demonstrated that intrathecal injection of GLP-1R agonist exenatide (100 ng) significantly enhanced mRNA levels of M2 microglial markers IL-10, IL-4, arginase-1 (Arg 1), and cluster of differentiation CD206 in both contralateral/ipsilateral spinal cords of neuropathic rats induced by tight ligation of L5/L6 spinal nerves (Wu et al., 2017a). Treatment of primary microglial cells with exenatide (0.1, 1, 10, 100, and 1,000 nM) for 2 h distinctly promoted mRNA levels of IL-10, IL-4, Arg 1, and CD206 dose-dependently, with half maximal effective concentration (EC_50_) values of 1.1, 1.4, 5.0, and 6.0 nM, respectively. In this study, morroniside did not change mRNA levels of marker proteins CD68 and inducible nitric oxide synthase (iNOS) for M1-type microglia, but significantly facilitated mRNA levels of marker proteins Agr1, C206, IL-4, and IL-10 for M2-type microglia (Figures 2A–F, *p* < 0.05, by using one-way ANOVA followed by *post hoc* Student–Newman–Keuls test). However, morroniside treatment failed to raise the expression of IL-10 protein significantly in astrocytes or neurons by ELISA (Figure 2G).

### GLP-1R/cAMP/PKA/p38β Pathway Mediated Morroniside-Induced Expression of IL-10 Protein in M2 Microglia

Mitogen-activated protein kinases (MAPKs) were a family of evolutionally conserved molecules including p38, extracellular signal-regulated kinase (ERK)1/2, and c-Jun N-terminal kinase (JNK)1/2 isoforms (Johnson and Lapadat, 2002). Their phosphorylation causally mediated the expression of both M1 and M2 microglia-specific proteins (Taves et al., 2013; Saraiva and O’Garra, 2010).

**FIGURE 3:**
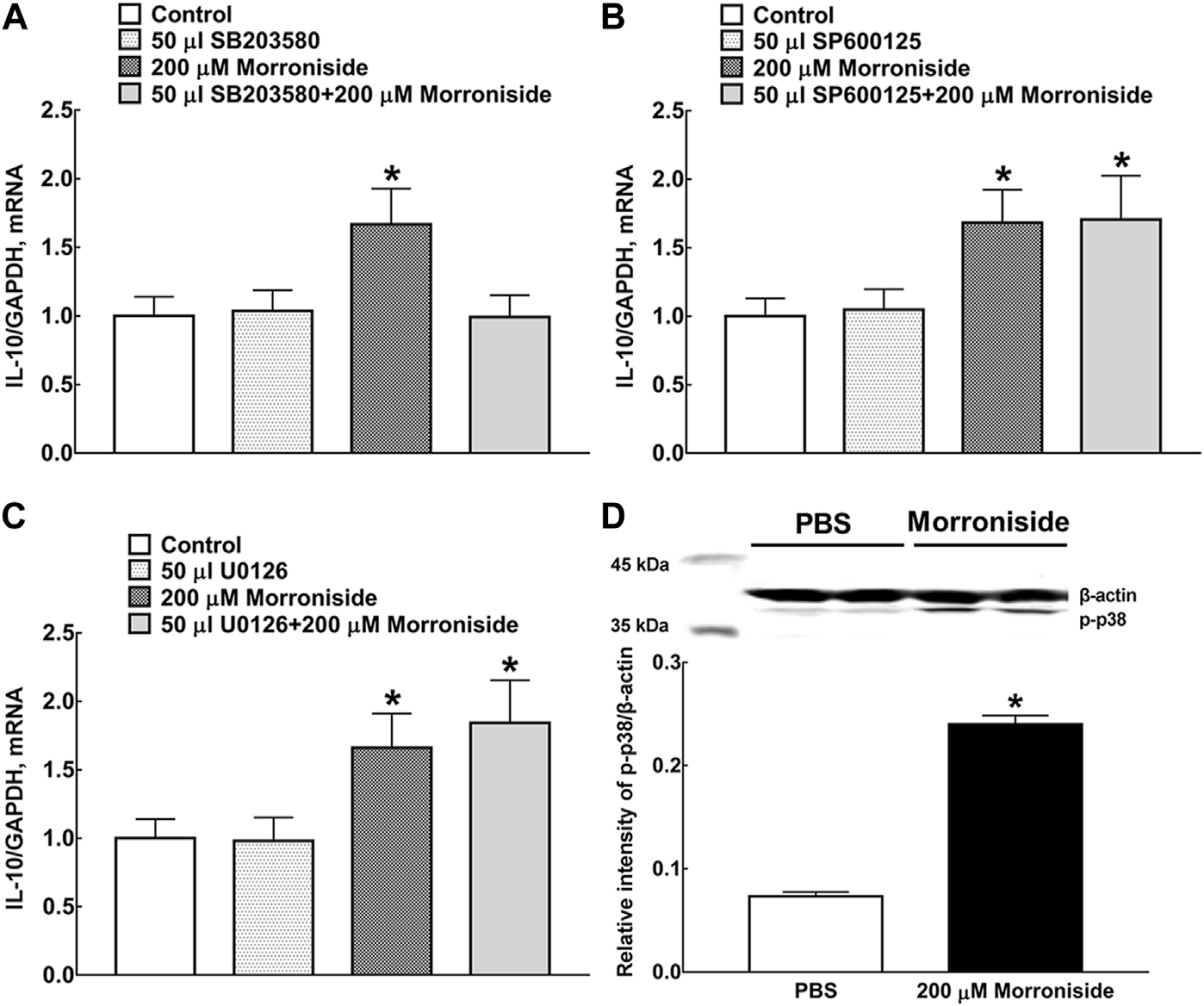
Effects of the selective p38 MAPK inhibitor SB203580 **(A)**, JNK MAPK inhibitor SP600125 **(B)**, and ERK1/2 MAPK inhibitor U0126 **(C)** on morroniside-induced IL-10 mRNA expression in primary microglia. **(D)** Effect of morroniside on p38 MAPK phosphorylation in primary microglia. The MAPK inhibitors were incubated 1 h before morroniside treatment. The culture medium and primary microglial cells were collected 2 h after morroniside incubation. Subsequently, the mRNA level of IL-10 was determined by RT-qPCR. The cellular experiments were repeated triply and all data were presented as means ± SEM (*n* = 3). *p < 0.05, significantly different from control group; ^#^ *p* < 0.05, significantly different from morroniside group; unpaired and two-tailed Student *t*-test.

Three small molecular drugs (the selective p38 MAPK inhibitor SB203580, ERK1/2 MAPK inhibitor U0126, and JNK MAPK inhibitor SP600125) were applied to identify the specific MAPK signaling pathway involved in morroniside-induced IL-10 expression in primary microglia (Figure 3A–C). The MAPK inhibitors were incubated 1 h before morroniside treatment. The culture medium and primary microglial cells were collected 2 h after morroniside incubation. The results showed that only SB203580 reversed the mRNA level of IL-10 induced by morroniside. Two hundred micromoles per liter of morroniside markedly promoted the p38 MAPK phosphorylation in protein level in primary microglia (Figure 3D, *p* < 0.05, by using unpaired and two-tailed Student *t*-test).

### Neuroprotective Effect of Morroniside in MCAO Mice

**FIGURE 4:**
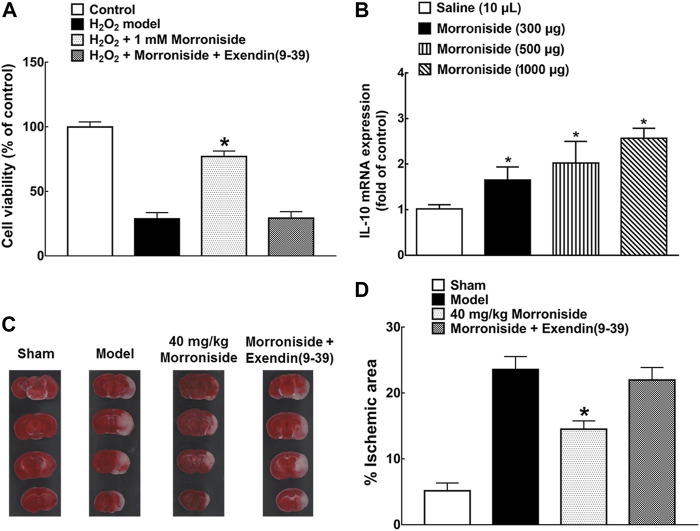
The neuroprotective effect of morroniside in MCAO mice. **(A)** GLP-1R mediated the effect of morroniside against cell oxidative damage; **(B)** IL-10 mRNA level in the cortical penumbra area in MCAO mice after multiple treatment of morroniside twice-daily by intracerebroventricular injection on day 3 after surgery; **(C, D)** the neuroprotective effect of morroniside was evaluated by intravenous injection in MCAO mice, while the GLP-1R antagonist exendin (9-39) completely inhibited its activity. The quantitative analysis was done by TTC staining (*n* = 6–8). The data were presented as mean ± SEM. Compared with model control group, *p < 0.05 denoted statistical significance by using one-way ANOVA and *post hoc* Student–Newman–Keuls tests in panel **(B)** or unpaired and two-tailed Student *t*-test in panels **(A)** and **(D)**.

Effect of morroniside against cell oxidative damage was first demonstrated in H_2_O_2_-induced N9 microglial cells by increase of 1.67 times on cell viability, which was completely blocked by GLP-1 receptor antagonist exendin (9-39) (Figure 4A, *p* < 0.05, by using unpaired and two-tailed Student *t*-test). The IL-10 mRNA level on day 3 after surgery in the cortical penumbra area in MCAO mice was also detected after multiple treatment of normal saline or morroniside twice-daily by intracerebroventricular injection. Morroniside (300, 500, 1,000 μg) enhanced the IL-10 mRNA level by 65, 101, and 149% (Figure 4B, *p* < 0.05, by using one-way ANOVA followed by *post hoc* Student–Newman–Keuls test). To evaluate the neuroprotective effect of morroniside by peripheral administration, 40 mg/kg morroniside was intravenously injected at 0 h after surgery and infarct size was evaluated 24 h later in MCAO mice by TTC staining (Figure 4C,D). The quantitative analysis showed that morroniside significantly reduced infarct size by 38.6%, which was completely prohibited by GLP-1 receptor antagonist exendin (9-39) (Figure 4D, *p* < 0.05, by using unpaired and two-tailed Student *t*-test). The intravenous injection was applied to assess the effect of morroniside by peripheral administration, which might prove its potential application in further clinical treatment. Besides, scientists also introduced intranasal administration as a promising strategy for active components from herbal medicine against ischemic stroke (Long et al., 2020).

## Discussion

GLP-1R exists in both the peripheral and central systems: 1) In the peripheral circulatory system, GLP-1R mostly exists in L-cells of the lower digestive tract, which can promote the secretion of insulin by pancreatic β cells, reduce the secretion of pancreas by pancreatic α cells, and simultaneously stimulate the proliferation and differentiation of pancreatic β-cells. 2) GLP-1R exists in the central nervous system, mainly expressed in microglia, but also a small amount in neurons. In the preclinical and clinical studies of Alzheimer’s disease and Parkinson’s disease, its neuroprotective functions were proved: GLP-1R agonists exerted anti-oxidant damage, neurotrophic properties and anti-apoptosis roughly through cAMP/PKA and PI3K/Akt signaling pathways, and effectively protect dopaminergic neurons and improve cognition and motor functions (Athauda and Foltynie, 2018; Batista et al., 2019).

Due to the differences in binding sites and binding methods with GLP-1R, different molecular structures of small molecule non-peptide GLP-1R agonists may perform different mechanisms of action (Zhao et al., 2020). We reported that a series of iridoid glycosides represented by geniposide methyl ester, geniposide, and morroniside effectively stimulated GLP-1R and resisted cell damage. Their dose-dependent activities were partially blocked by the GLP-1R antagonist exendin (9-39) (Gong et al., 2014b; Zhu et al., 2014; Xu et al., 2017). The anti-apoptotic effect of morroniside against the exposure of H_2_O_2_ or Aβ(1–42) was reported and explained as not only preventing JNK and p38 MAPK phosphorylation but also suppressing its related upstream signaling (Chen et al., 2018). Our previous study first elucidated the straightforward casual association between morroniside as GLP-1R agonist and anti-apoptosis in H_2_O_2_-induced N9 cell death (Xu et al., 2017). Plenty of evidence indicated that iridoid glycosides, as small molecule non-peptide GLP-1R agonists, were an optional treatment strategy against ischemic stroke.

**FIGURE 5:**
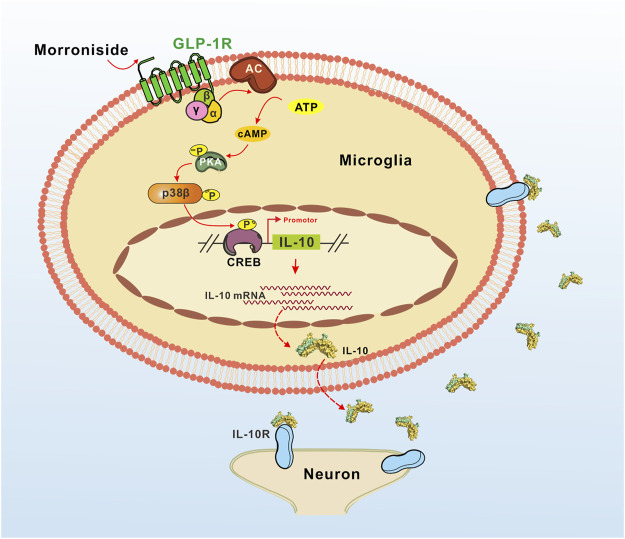
Illustration of the proposed IL-10 expression induced by GLP-1R agonist morroniside in MCAO mice. As the main pathway of GLP-1R, the cAMP/PKA/p38β signal cascade mediated IL-10 expression and secretion. According to our viewpoints in general, the secreted IL-10 then might play a neuroprotective effect by anti-inflammation and anti-apoptosis.

The potential mechanism of the proposed IL-10 expression induced by GLP-1R agonist morroniside is illustrated in Figure 5. As the main pathway of GLP-1R, the cAMP/PKA/p38β signal cascade mediated IL-10 expression and secretion. IL-10 was believed to inhibit the expression of neuroinflammtory cytokine TNF-α, IL-1β, and IL-6, and to induce anti-inflammatory elements like Bcl3 and Socs3 to further prevent NF-κB pathway (Ji et al., 2002; Milligan et al., 2012). In general, our results indicated that GLP-1R agonist morroniside might play a neuroprotective effect by inducing M2 polarization, and cAMP/PKA/p38β pathway might mediate morroniside-induced expression of IL-10 protein in M2 microglia.

## Data Availability

The original contributions presented in the study are included in the article/supplementary materials. Further inquiries can be directed to the corresponding author.
